# Novel juxtamembrane *FLT3* in-frame insertion in a child with refractory AML: a case report of durable remission following allogeneic HSCT and *FLT3* inhibitor therapy

**DOI:** 10.3389/fonc.2026.1852452

**Published:** 2026-09-25

**Authors:** Abdul Ali Peer-Zada, Mohammed Al-Rimawi, Farah Malaeb, Tariq Faquih, Monther Alhamdoosh, Abdulrahman Alsultan, Walid Ballourah

**Affiliations:** 1 Molecular Genetics Section, Pathology and Clinical Laboratory Medicine Administration, King Fahad Medical City, Riyadh, Saudi Arabia; 2 College of Medicine, Alfaisal University, Riyadh, Saudi Arabia; 3 Department of Pediatric Hematology and Oncology, King Fahad Medical City, Riyadh, Saudi Arabia; 4 Computational Sciences Department, Genomic Medicine Centre of Excellence, King Faisal Specialist Hospital and Research Center, Riyadh, Saudi Arabia; 5 Department of Pediatrics, College of Medicine, King Saud University, Riyadh, Saudi Arabia; 6 Department of Pediatric Hematology/Oncology, King Faisal Specialist Hospital and Research Center, Riyadh, Saudi Arabia

**Keywords:** acute myeloid leukemia, allogeneic hematopoietic stem cell transplantation, FLT3, melphalan, next-generation sequencing, sorafenib, total body irradiation

## Abstract

**Background:**

*FLT3* gene mutations, particularly internal tandem duplications (*FLT3*-ITD), are present in approximately 25%–30% of patients with acute myeloid leukemia (AML) and are important drivers of leukemogenesis; less frequently, *FLT3* mutations occur as tyrosine kinase domain (TKD) variants. Non-canonical variants, such as juxtamembrane domain (JMD) in-frame insertions, are rare and their pathogenic mechanisms and therapeutic sensitivities are less defined.

**Case description:**

We report a 13-year-old female patient with refractory AML harboring a novel in-frame insertion in the *FLT3* JMD NC_000013.11(NM_004119.3):c.1780_1781insGGGTCCCTCCGCCTACCC; p.(Phe594delinsTrpValProProProThrLeu) identified by next-generation sequencing (NGS). The disease was resistant to standard intensive chemotherapy but showed marked sensitivity to the *FLT3* inhibitor sorafenib. Following relapse upon sorafenib discontinuation due to cardiac toxicity, the patient underwent allogeneic hematopoietic stem cell transplantation (HSCT) while in active disease (M3 marrow). A sequential myeloablative conditioning regimen consisting of melphalan, fludarabine, and fractionated total body irradiation (TBI-8 Gray) was employed. Post-transplant, sorafenib maintenance was resumed for 2 years.

**Outcomes:**

The patient achieved sustained complete remission and remains disease-free 63 months post-HSCT, despite significant pre-transplant comorbidities including fungal pneumonia and a pulmonary artery pseudo-aneurysm.

**Conclusion:**

This case suggests that non-canonical *FLT3* JMD insertions may be actionable molecular alterations and highlights the need for further investigation of *FLT3* inhibitor efficacy and the therapeutic relevance of these rare mutations in AML.

## Introduction

The *FLT3* (FMS-like tyrosine kinase 3) gene encodes a class III receptor tyrosine kinase essential for hematopoietic stem cell proliferation and survival (1). In acute myeloid leukemia (AML), *FLT3* mutations occur in approximately 25%–30% of cases, with internal tandem duplications (ITDs) in exons 14 and 15 and point mutations in the tyrosine kinase domain (TKD) being the most prevalent (2, 3). These mutations disrupt the auto-inhibitory function of the juxtamembrane domain (JMD) or the activation loop, leading to constitutive, ligand-independent activation of downstream signaling pathways such as PI3K/AKT, RAS/MAPK, and STAT5 (4, 5). *FLT3* mutations in AML confer markedly worse prognosis; the Japanese Childhood AML Cooperative Study (AML-99) reported a 3-year overall survival of 35.3% in *FLT3*-ITD-positive patients versus 84.3% in *FLT3*-wild-type patients (6). These outcomes are substantially improved by the addition of targeted *FLT3* inhibitors to treatment regimens (7, 8). While the prognostic impact of canonical *FLT3*-ITD and TKD mutations is well-established, non-canonical variants, including small in-frame insertions or deletions within the JMD, remain poorly characterized (9).

We present a unique case of pediatric AML driven by a novel *FLT3* JMD in-frame insertion NC_000013.11(NP_004110.2):p.(Phe594delinsTrpValProProProThrLeu). This case illustrates the possible pathogenicity of the variant, the clinical response to sorafenib, and the successful application of an intensified sequential conditioning regimen (Melphalan/Fludarabine/TBI-8) for allogeneic hematopoietic stem cell transplantation (HSCT) in the setting of active disease.

## Case description

### Patient presentation and diagnostic assessment

A 13-year-old female patient presented with a history of fatigue, petechiae, splenomegaly, and bilateral cervical lymphadenopathy. Laboratory investigations revealed leukocytosis, anemia, and thrombocytopenia, with 20% blasts on the peripheral smear. Bone marrow (BM) biopsy showed 100% cellularity with infiltration by immature myeloid cells. Flow cytometry confirmed a CD45^+^ population (82%) expressing CD34, CD38-dim, CD13, CD7, CD15, CD117, CD58, and CD11c, but negative for CD33, CD11b, CD64, CD4, MPO, and CD3. Cytogenetics revealed a normal karyotype, and FISH was negative for common AML translocations.

PCR-based screening for *FLT3*-ITD and NPM1 was negative. However, next-generation sequencing (NGS) (Centogene, Germany) utilizing a 35-gene myeloid panel identified a novel in-frame insertion in *FLT3* exon 14: NC_000013.11(NM_004119.3):c.1780_1781insGGGTCCCTCCGCCTACCC; NC_000013.11(NP_004110.2):p.(Phe594delinsTrpValProProProThrLeu). The variant was not present in gnomAD, Online Mendelian Inheritance in Man (OMIM), Clinvar, UniProt, The Cancer Genome Atlas (TCGA), or the local Saudi genomic databases. In addition, to verify that the sequence is not derived from adjacent *FLT3* regions, we examined the full sequence of *FLT3* (NCBI Reference Sequence: NG_007066.1) and screened for the presence of the inserted sequence GGGTCCCTCCGCCTACCC. Based on this, we did not find this sequence to be present anywhere in the FTL3 genomic region. Other *FLT3*-ITD variants in the same region reported to date with HGVS-based nomenclature are shown for comparison (**Table 1**).

**Table 1:** Current case and previously reported *FLT3*-ITD mutations in the same genomic location.

Position (*FLT3*: NM_004119.2)	*FLT3*-ITD mutation(nucleotide)	*FLT3*-ITD mutation (amino acid)	COSMIC mutation ID
594	c.1780_1781ins GGGTCCCTCCGCCTACCC	p.D593_F594insWVPPPTL	Current case
594	c.1780_1781insCAGAGAATATGAATATGATCT	p.D593_F594insSENMNMI	COSM10189273
594	c.1780_1781insCCCCGAATGAGTACTTCTACGTTGATT	p.D593_F594insSPNEYFYVD	COSM10112855
594	c.1780_1781insCCCCGGAAGATAATGAGTACTTCTACGTTGATT	p.D593_F594insSPEDNEYFYVD	COSM1317908
594	c.1780_1781insGGAGAGAGAGAGATAATGATAATGAGTACTTCTACGTTGATT	p.D593_F594insWRERDNDNEYFYVD	COSM5879541
594	c.1780_1781insGGGGGTACTTCTACGTTGATT	p.D593_F594insWGYFYVD	COSM19951

### Therapeutic intervention and clinical course

The patient was treated according to the Children’s Oncology Group AAML0531 protocol. Induction with ADE (cytarabine, daunorubicin, and etoposide) failed to clear the blasts (97% residual). Subsequent salvage with FLAG (fludarabine, cytarabine, and G-CSF) also resulted in persistent disease (90% blasts). Given the NGS findings, the *FLT3* inhibitor sorafenib was added to clofarabine and high-dose cytarabine. This targeted approach achieved complete morphological remission (2% blasts). Unfortunately, the patient developed severe complications, including fungal pneumonia with a life-threatening pulmonary artery branch pseudo-aneurysm and cardiac dysfunction. Sorafenib was discontinued due to cardiotoxicity, leading to an immediate relapse. Re-challenge with chemotherapy (clofarabine/high-dose cytarabine) without sorafenib failed to regain remission. Other *FLT3* inhibitors could not be administered because of limited availability and lack of approval for pediatric use by national regulatory authorities.

Over several months, the patient received additional salvage cycles (including azacitidine/low-dose sorafenib, azacitidine/venetoclax, and TVTC: topotecan, vinorelbine, clofarabine, and thiotepa) (10), with the aim of controlling disease activity while allowing for pulmonary fungal infection control and cardiac function recovery. BM blasts and peripheral blasts persisted (**Figure 1**).

**Figure 1:**
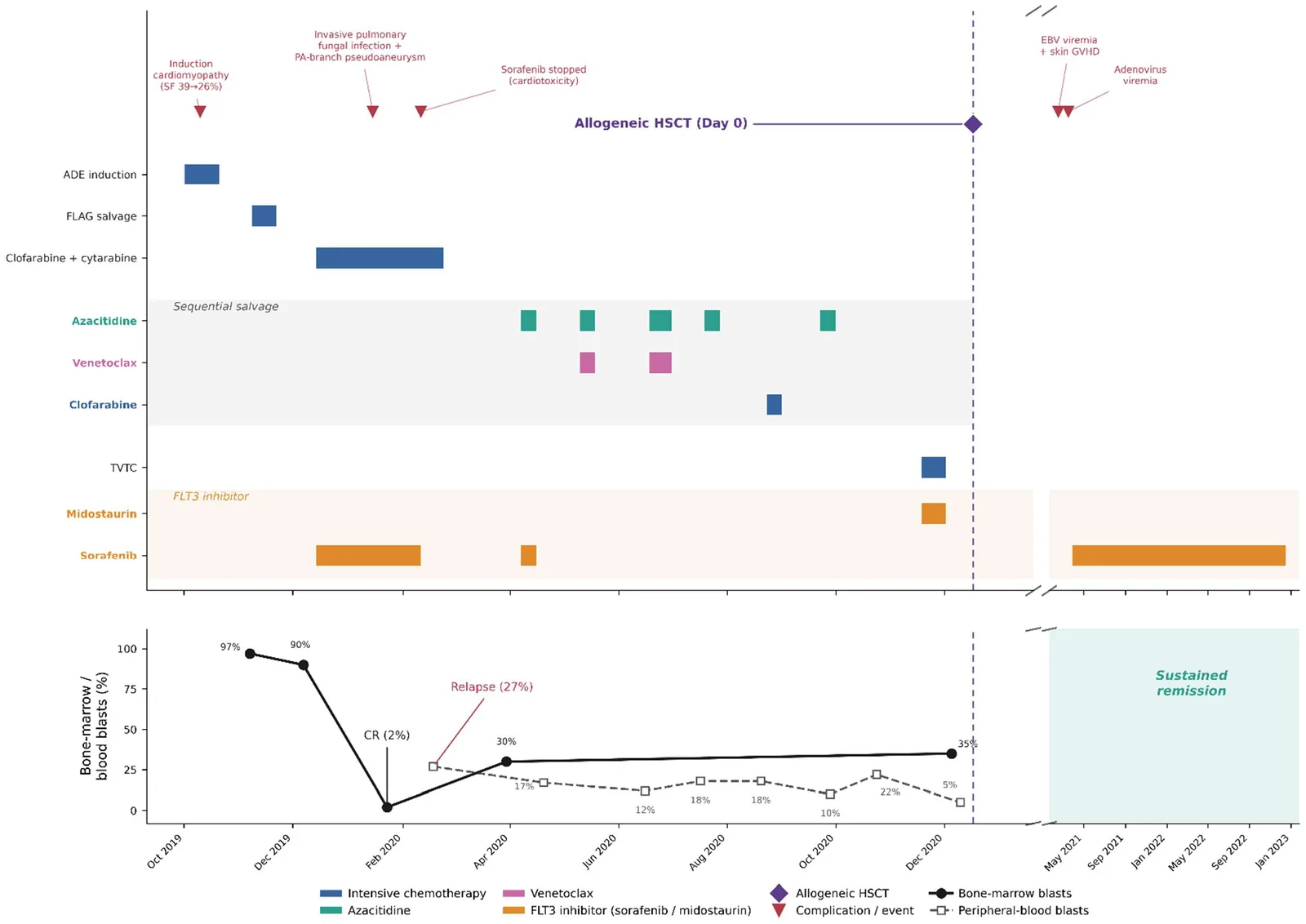
Timeline of treatment modalities, response, and complications throughout the patient’s journey.

### Allogeneic HSCT and conditioning

Following radiologic stabilization of the invasive pulmonary fungal infection and improvement in cardiac function (shortening fraction 30%), the patient proceeded to a 10/10 HLA-matched sibling donor allogeneic HSCT despite persistent active leukemia. Pre-transplant BM evaluation demonstrated 35% blasts without additional cytogenetic or molecular abnormalities. At transplantation, the white blood cell count was 12 × 10^9^/L, platelet count was 45 × 10^9^/L, and 5% circulating blasts were present. A sequential myeloablative conditioning regimen consisting of melphalan (140 mg/m², day −11), fludarabine (30 mg/m²/day, days −6 to −3), and fractionated total body irradiation (2 Gy twice daily, days −2 to −1; total dose 8 Gy) was administered (11). Peripheral blood stem cells were infused at a CD34^+^ cell dose of 8 × 10^6^/kg. Graft-versus-host disease (GvHD) prophylaxis consisted of cyclosporine and methotrexate (10 mg/m² on days +1, +3, and +6). Immunosuppression tapering was initiated on day +45 to facilitate a graft-versus-leukemia effect through controlled alloreactivity.

### Follow-up and outcomes

Successful neutrophil and platelet engraftment occurred by the third week. Full donor chimerism (100%) was achieved and maintained at days +30, +60, and +100. Post-transplant complications included BK virus-associated hemorrhagic cystitis and EBV/adenovirus viremia, all of which were managed successfully.

Sorafenib maintenance was initiated at 100 mg daily and continued for 2 months. After confirmation of sustained cardiac stability, the dose was increased to 150 mg daily for 2 months and subsequently to 200 mg daily, which was continued until 2 years post-transplant. Sorafenib was then discontinued. At 63 months after allogeneic HSCT, the patient remains in complete remission with 100% donor chimerism.

### Protein structure and impact

To assess and visualize the structural alteration to the FTL3 protein caused by our reported mutation, we performed several *in silico* protein structure predictions using SWISS-model (12, 13). The main model used our reported mutation (Phe594delinsTrpValProProProThrLeu) and the wild-type protein structure was used as the alignment template (protein structure P36888.1 for FTL3). Similarly, we performed the same modeling steps using other mutations reported in literature: (i) NC_000013.11(NP_004110.2):p.(F594delinsSL) (14), a smaller insertion–deletion mutation with a different amino acid composition than our reported mutation; (ii) NC_000013.11(NP_004110.2):p.(Tyr597_Glu598insAspValAspPheArgGluTyr) (15), an established ITD mutation; and (iii) NC_000013.11(NP_004110.2): p.(Phe594_Asp600delinsSerProProProHis), a deletion of phenylalanine and an insertion of several proline amino acids, similar to our reported mutation. Mutant structures were superimposed against the original wild-type *FLT3* protein (P36888.1) based on the upstream and downstream amino acid sequences surrounding the mutation site (i.e., 580–590 and 596–606 of the wild-type protein). Results of the homology modeling and comparisons are presented in **Figures 2**, **3**.

**Figure 2:**
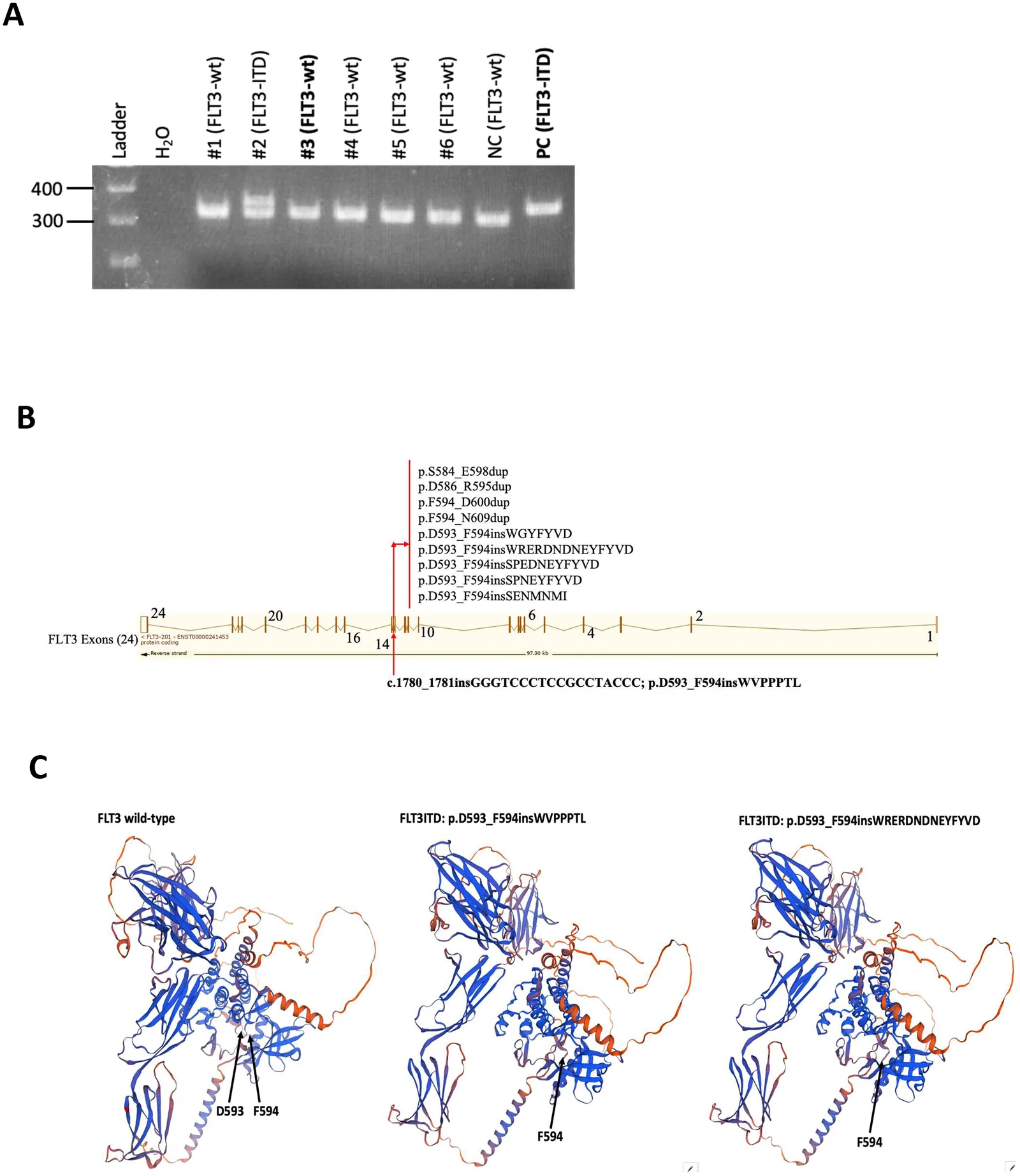
**(A)** PCR followed by gel electrophoresis for the detection of previously reported *FLT3*-ITD using a commercially available kit (invivoscribe, USA). Lanes are marked as follows: ladder, 100-base-pair (bp) marker; water control; #1 to #6 various patient specimen with #3 (current case); NC wild-type *FLT3* (*FLT3*-wt); PC positive control for *FLT3*-ITD. The expected size range is marked between 300 and 400 bp. Note that the patient specimen with an ITD shows two distinct bands (#2). **(B)** Exon map of *FLT3* gene with the novel variant identified in exon 14 and previously reported variants (ITD). Detailed exon map of *FLT3* gene in reverse direction with the solid bars representing exons (right to left) and arrows showing the variants at specific exons. The number beside the gene name represents total number of exons in *FLT3* gene. **(C)** 3D structures obtained for wild-type *FLT3* gene and the ITD mutants (current case and one previously reported) using the Swissmodel (swissmodel.expasy.org) prediction.

**Figure 3:**
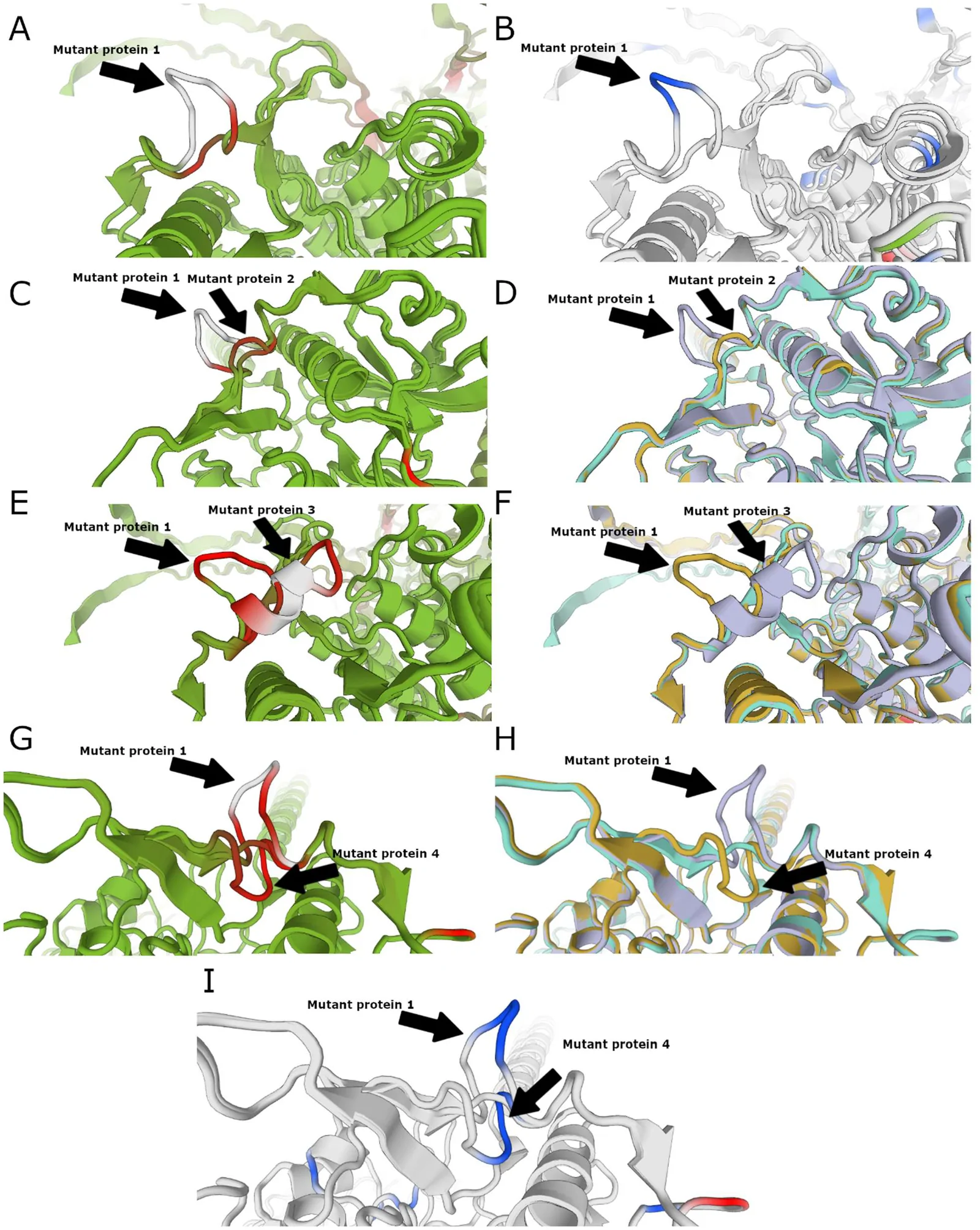
3D structure superposition between the wild *FLT3* protein and mutations affecting the Phe594 juxtamembrane (JM) domain region, specifically JM-Z, in Swiss-Model: **(A)** Comparisons between the wild-type protein (P36888-F1) and the predicted protein structure with our reported mutation. Green indicates consistency between the sequences and structure in Ensemble. Red indicates poor consistency. White indicates no consistency. The white chain is our mutated sequence. **(B)** The same superposed structures as A, the blue color highlights represent the Proline sequences. **(C)** Additional superposition of a third protein structure (mutant protein 2). Red indicates poor consistency with the Ensemble sequence. **(D)** Same proteins as **(C)** Cyan indicates the wild-type protein. Light purple represents our predicted protein. Yellow represents mutant protein 2. **(E)** Superposition of the wild-type protein, our predicted protein, and mutant protein 4, which has an internal tandem repeat (ITD) (Clinvar ID: VCV000219096). Red indicates the inconsistency between the sequence and the Ensemble sequence. Notably, internal tandem repeat creates a ribbon structure not found in our reported mutated protein. **(F)** Same as **(E)** Cyan indicates the wild-type protein. Yellow indicates our predicted protein. Light purple indicates mutant protein 4. **(G)** Superposition of the wild-type protein, our protein, and mutant protein 4. The colors represent consistency between the amino acid sequences. **(H)** Same superposition as **(G)** Light purple represents our protein mutation and yellow represents mutant protein 4. **(I)** Same superposition as **(H)** and **(G)**. The blue color highlights represent the Proline sequences in our reported protein and mutant protein 4. The arrows and their labels point to the specific mutant proteins: Mutant protein 1: Phe594delinsTrpValProProProThrLeu; Mutant protein 2: Phe594delinsSerLeu; Mutant protein 3: Tyr597_Glu598insAspValAspPheArgGluTyr; Mutant protein 4: Phe594_Asp600delinsSerProProProHis.

## Discussion

This report characterizes a novel in-frame insertion in the *FLT3* JMD NC_000013.11(NP_004110.2):p.(Phe594delinsTrpValProProProThrLeu) and highlights its role as a pathogenic driver in AML. According to ACMG criteria, the variant is classified as likely pathogenic based on its absence in population databases (PM2), protein length change in a non-repeat region (PM4), and the highly specific clinical phenotype of refractory AML with rapid response to *FLT3* inhibition (PP4) (16).

### Possible pathogenicity of the *FLT3* JMD insertion

The JMD of *FLT3* normally maintains the kinase in an inactive state by blocking the ATP-binding pocket. Structural disruption of this region, typically via ITDs, promotes ligand-independent dimerization and constitutive activation (17). The identified insertion involves codon Phe594, a residue within the JMD known to be critical for autoinhibition. Similar non-canonical JMD insertions have been shown *in vitro* to induce autonomous receptor signaling and cellular transformation (18). In our patient, the strict therapeutic dependence on sorafenib—evidenced by remission only upon its addition and immediate relapse upon its withdrawal—provides clinical evidence supporting an activating effect of this novel mutation.

### *In silico* prediction of the impact of *FLT3* JMD insertion on JMD protein structure

Based on our homology modeling observations, it is evident that our reported mutation alters the JM-Z domain. In addition, the proline chain inserted is predicted to have a different structure than previously reported ITD mutation and even non-ITD mutation with a similar proline insertion. Given the clinical observations when using *FLT3* inhibitors and considering that the publicly reported mutations have been confirmed to have moderate to high impact and are likely oncogenic, we can hypothesize that our reported mutation is likely to be affecting the JMD domain and possibly disrupting the JM-Z/N-lobe interaction, similar to other reported *FLT3* mutations but with different structural conformation.

### HSCT outcomes in AML with active disease: evolving evidence

Allogeneic HSCT for childhood AML is associated with significantly inferior outcomes when performed in the presence of active disease, with reported overall survival ranging from 18% to 44% compared with 56%–71% for patients transplanted in complete remission (19–21). Doherty et al. demonstrated a 2.4-fold higher relapse incidence among children transplanted with active disease [sub-distribution hazard ratio (HR) 2.42, *p* = 0.016], while an EBMT registry study reported a cumulative relapse incidence of 45.1% in patients with active t (6, 9) AML compared with 18.2% in those transplanted in first complete remission (21). Similarly, disease not in remission before HSCT was the only independent predictor of inferior overall survival in a multicenter cohort (HR 2.27, *p* < 0.05) (20). In a United Kingdom cohort of children with refractory AML, a pre-transplant BM blast burden ≤30% was associated with significantly better 5-year leukemia-free survival than >30% blasts (52% vs. 27%) (22).

Although prospective pediatric data are lacking, the adult ASAP trial demonstrated that immediate allogeneic HSCT without prior remission–induction chemotherapy achieved survival comparable to conventional salvage therapy while reducing treatment-related toxicity and shortening the time to transplantation (23). Long-term follow-up further identified ELN genetic risk, age, and comorbidity burden—not remission status at transplantation—as the primary determinants of survival (24). Although these findings cannot be directly extrapolated to pediatric AML, they support the concept that failure to achieve morphological remission should not automatically preclude HSCT in carefully selected patients with refractory disease.

Our patient underwent HSCT for refractory AML with 35% BM blasts and multiple significant pre-transplant comorbidities, including cardiomyopathy and a pulmonary artery aneurysm, placing her at an exceptionally high risk of post-transplant relapse and mortality. Nevertheless, allogeneic HSCT followed by post-transplant sorafenib maintenance resulted in sustained leukemia-free survival exceeding 5 years.

### Conditioning regimen for allogenic HSCT

Melphalan (140 mg/m²) combined with fludarabine and total body irradiation (8 Gy) provided potent cytoreduction in this case. High-dose melphalan is an effective myeloablative agent for refractory AML, and its combination with TBI has been associated with enhanced blast clearance and reduced relapse in relapsed/refractory disease (11, 25). Despite significant pre-transplant comorbidities, the patient tolerated the regimen, achieved durable engraftment, and remained leukemia-free for more than 5 years after HSCT.

### The role of post-transplant TKI maintenance

Relapse remains the primary cause of failure after HSCT for *FLT3*-mutated AML. Recent evidence supports the use of *FLT3* inhibitors like sorafenib as maintenance therapy post-HSCT to reduce relapse rates and improve overall survival (26, 27). Our patient’s sustained remission (63 months) following 2 years of sorafenib maintenance reinforces the utility of this strategy, particularly for variants that exhibit high TKI sensitivity.

### Strengths and limitations

A key strength of this report is the integration of longitudinal clinical follow-up exceeding 5 years, comprehensive molecular characterization, *in silico* structural modeling, and clinical response data related to a novel FLT3 JMD insertion. The concordance between homology-based structural predictions, the observed sensitivity to *FLT3* inhibition, and the durable post-transplant remission provides convergent evidence supporting the putative pathogenicity and therapeutic relevance of this variant. A limitation is the absence of direct *in vitro* functional studies, such as phospho-*FLT3* signaling assays or cellular transformation experiments, to definitively characterize the molecular consequences of the mutation, although the computational and clinical findings suggest an activating effect.

### Patient perspective

At the time of writing, the patient has achieved sustained remission and has returned to normal daily activities, including attending college. Both the patient and her family express deep gratitude for this unexpected and highly favorable outcome in what was an exceptionally challenging clinical course. Prior to this, the family had largely lost hope, particularly after the disease proved refractory to many lines of therapy and following multiple consultations with international experts, all of whom indicated an extremely poor prognosis with a near-zero likelihood of cure. Currently, the patient is thriving, with a good quality of life, and the family is able to enjoy her recovery and reintegration into normal life.

## Data Availability

The original contributions presented in the study are included in the article, further inquiries can be directed to the corresponding author.

## References

[1] Rosnet O Matteï MG Marchetto S Birnbaum D . Isolation and chromosomal localization of a novel FMS-like tyrosine kinase gene. Genomics. (1991) 9:380–5. doi: 10.1016/0888-7543(91)90270-o 2004790

[2] Griffith J Black J Faerman C Swenson L Wynn M Lu F . The structural basis for autoinhibition of *FLT3* by the juxtamembrane domain. Mol Cell. (2004) 13:169–78. doi: 10.1016/s1097-2765(03)00505-7 14759363

[3] Arber DA Orazi A Hasserjian R Thiele J Borowitz MJ Le Beau MM . The 2016 revision to the World Health Organization classification of myeloid neoplasms and acute leukemia. Blood. (2016) 127:2391–405. doi: 10.1182/blood-2016-03-643544 27069254

[4] Bystrom R Levis MJ . An update on *FLT3* in acute myeloid leukemia: Pathophysiology and therapeutic landscape. Curr Oncol Rep. (2023) 25:369–78. doi: 10.1007/s11912-023-01389-2 36808557

[5] Döhner H Estey E Grimwade D Amadori S Appelbaum FR Büchner T . Diagnosis and management of AML in adults: 2017 ELN recommendations from an international expert panel. Blood. (2017) 129:424–47. doi: 10.1182/blood-2016-08-733196 27895058 PMC5291965

[6] Shimada A Taki T Tabuchi K Taketani T Hanada R Tawa A . Tandem duplications of MLL and *FLT3* are correlated with poor prognoses in pediatric acute myeloid leukemia: A study of the Japanese childhood AML Cooperative Study Group. Pediatr Blood Cancer. (2008) 50:264–9. doi: 10.1002/pbc.21318 17763464

[7] Oñate G Pratcorona M Garrido A Artigas-Baleri A Bataller A Tormo M . Survival improvement of patients with *FLT3* mutated acute myeloid leukemia: results from a prospective 9 years cohort. Blood Cancer J. (2023) 13:69. doi: 10.1038/s41408-023-00839-1 37147301 PMC10162955

[8] Matsukawa T Onozawa M Kondo T Kanaya M Hidaka D Ota S . *FLT3* inhibitors and hematopoietic cell transplantation prolong survival in patients with *FLT3*-ITD-positive AML. Ann Hematol. (2024) 103:5333–40. doi: 10.1007/s00277-024-06125-9 10.1007/s00277-024-06125-9 39614924

[9] Luthra R Patel KP Reddy NG Haghshenas V Routbort MJ Harmon MA . Next-generation sequencing-based multigene mutational screening for acute myeloid leukemia using MiSeq: applicability for diagnostics and disease monitoring. Haematologica. (2014) 99:465–73. doi: 10.3324/haematol.2013.093765 24142997 PMC3943309

[10] Ramaswamy K Steinherz PG Agrawal AK Forlenza CJ Mauguen A Roshal M . Clofarabine with topotecan, vinorelbine, and thiotepa reinduction regimen for children and young adults with relapsed AML. Blood Adv. (2022) 6:2688–94. doi: 10.1182/bloodadvances.2021005753 35008101 PMC9043926

[11] Steckel NK Groth C Mikesch J Trenschel R Ottinger H Kordelas L . High‐dose melphalan‐based sequential conditioning chemotherapy followed by allogeneic haematopoietic stem cell transplantation in adult patients with relapsed or refractory acute myeloid leukaemia. Br J Haematol. (2018) 180:840–53. doi: 10.1111/bjh.15137 29468631

[12] Bienert S Waterhouse A de Beer TAP Tauriello G Studer G Bordoli L . The SWISS-MODEL Repository-new features and functionality. Nucleic Acids Res. (2017) 45:D313–9. doi: 10.1093/nar/gkw1132 27899672 PMC5210589

[13] Waterhouse A Bertoni M Bienert S Studer G Tauriello G Gumienny R . SWISS-MODEL: homology modelling of protein structures and complexes. Nucleic Acids Res. (2018) 46:W296–303. doi: 10.1093/nar/gky427 29788355 PMC6030848

[14] Reindl C Bagrintseva K Vempati S Schnittger S Ellwart JW Wenig K . Point mutations in the juxtamembrane domain of *FLT3* define a new class of activating mutations in AML. Blood. (2006) 107:3700–7. doi: 10.1182/blood-2005-06-2596 16410449

[15] Abu-Duhier FM Goodeve AC Wilson GA Gari MA Peake IR Rees DC . *FLT3* internal tandem duplication mutations in adult acute myeloid leukaemia define a high-risk group. Br J Haematol. (2000) 111:190–5. doi: 10.1046/j.1365-2141.2000.02317.x 11091200

[16] Richards S Aziz N Bale S Bick D Das S Gastier-Foster J . Standards and guidelines for the interpretation of sequence variants: a joint consensus recommendation of the American College of Medical Genetics and Genomics and the Association for Molecular Pathology. Genet Med. (2015) 17:405–24. doi: 10.1038/gim.2015.30 25741868 PMC4544753

[17] Zhang W Konopleva M Shi YX McQueen T Harris D Ling X . Mutant *FLT3*: a direct target of sorafenib in acute myelogenous leukemia. J Natl Cancer Inst. (2008) 100:184–98. doi: 10.1093/jnci/djm328 18230792

[18] Thiede C Steudel C Mohr B Schaich M Schäkel U Platzbecker U . Analysis of *FLT3*-activating mutations in 979 patients with acute myelogenous leukemia: association with FAB subtypes and identification of subgroups with poor prognosis. Blood. (2002) 99:4326–35. doi: 10.1182/blood.v99.12.4326 12036858

[19] Doherty E Redell M Sasa G Yassine K John T Craddock J . Outcomes after allogeneic hematopoietic stem cell transplantation for pediatric acute myeloid leukemia in the contemporary era. Biol Blood Marrow Transplant. (2020) 26:Supplement S100. doi: 10.1016/j.bbmt.2019.12.60242574925

[20] Bhat S Jain S Yadev S Nirmal G Kalra G Daruvala S . Hematopoietic stem cell transplant for acute myeloid leukaemia in children and adolescents: multicentric experience from India. Blood. (2024) 144:7434. doi: 10.1182/blood-2024-207098

[21] Andreozzi F Galimard JE Maertens J Aljurf M Clesham K Sanz J . Outcomes of 544 Patients with t(6;9)/DEK::NUP214 acute myeloid leukemia undergoing allogeneic stem cell transplantation: an EBMT study on behalf of the acute leukemia working party (ALWP) and the pediatric diseases working party (PDWP). Bone Marrow Transplant. (2026) 61:838–46. doi: 10.1038/s41409-026-02857-6 42020760

[22] O’Hare P Lucchini G Cummins M Veys P Potter M Lawson S . Allogeneic stem cell transplantation for refractory acute myeloid leukemia in pediatric patients: the UK experience. Bone Marrow Transplant. (2017) 52:825–31. doi: 10.1038/bmt.2017.3 28218757

[23] Stelljes M Middeke JM Bug G Wagner-Drouet EM Müller LP Schmid C . Remission induction versus immediate allogeneic haematopoietic stem cell transplantation for patients with relapsed or poor responsive acute myeloid leukaemia (ASAP): a randomised, open-label, phase 3, non-inferiority trial. Lancet Haematol. (2024) 11:e324–35. doi: 10.1016/S2352-3026(24)00065-6 38583455

[24] Stelljes M Middeke JM Bug G Wagner-Drouet EM Müller LP Schmid C . Disease risk but not remission status determines transplant outcomes in AML: long-term outcomes of the ASAP trial. Blood. (2025) 146:2293–305. doi: 10.1182/blood.2025028730 40737595 PMC12824691

[25] Ronnacker J Urbahn MA Reicherts C Kolloch L Berning P Call S . Blast clearance after high dose-melphalan conditioning for allogeneic stem cell transplantation in relapsed and refractory acute myeloid leukemia. Blood. (2023) 142:4911. doi: 10.1182/blood-2023-180723

[26] Antar A Kharfan-Dabaja MA Mahfouz R Bazarbachi A . Sorafenib maintenance appears safe and improves clinical outcomes in *FLT3*-ITD acute myeloid leukemia after allogeneic hematopoietic cell transplantation. Clin Lymphoma Myeloma Leuk. (2015) 15:298–302. doi: 10.1016/j.clml.2014.12.005 25550214

[27] Xuan L Wang Y Huang F Fan Z Xu Y Sun J . Sorafenib maintenance in patients with *FLT3*-ITD acute myeloid leukaemia undergoing allogeneic haematopoietic stem-cell transplantation: an open-label, multicentre, randomised phase 3 trial. Lancet Oncol. (2020) 21:1201–12. doi: 10.1016/S1470-2045(20)30455-1 32791048

